# Mandarin Chinese *wh*-in-situ argument–adjunct asymmetry in island sensitivity: Evidence from a formal judgment study

**DOI:** 10.3389/fpsyg.2022.954175

**Published:** 2022-09-09

**Authors:** Qilin Tian, Myung-Kwan Park, Xiaodong Yang

**Affiliations:** ^1^School of English Studies, Zhejiang International Studies University, Hangzhou, China; ^2^Department of English, Dongguk University, Seoul, South Korea; ^3^College of Foreign Languages, Zhejiang University of Technology, Hangzhou, China

**Keywords:** island, *wh*-in-situ, argument–adjunct asymmetry, experimental syntax, acceptability judgment, pragmatic constraint

## Abstract

Unlike adjunct *wh’s*-in-situ, argument *wh’s*-in-situ do not seem to be subject to island constraints in Chinese and other East Asian languages. This difference in island sensitivity between argument and adjunct *wh’s*-in-situ is known as argument–adjunct asymmetry in the theoretical literature. Recently, this long-established asymmetry is challenged by a formal judgment study. It was claimed in the study that this asymmetry is an illusion and both argument and adjunct *wh’s*-in-situ are subject to island constraints. The present study demonstrates that such a claim is not convincing because it is based on problematic experimental design. We designed two experiments to test the island effects on Chinese *wh’s*-in-situ. The results reaffirm that the argument–adjunct asymmetry in Chinese *wh’s*-in-situ is indeed present, contrary to the findings of previous formal judgment study, and they also corroborate our assumption that when object *wh’s*-in-situ like *shénme* ‘what’ are located inside a relative clause, they are subject to a pragmatic constraint, suggesting that the VP (formed by a verb and its wh-object) in the relative clause tends to describe the prominent/salient feature of the relativized nominal head.

## Introduction

In English and many other languages of Indo-European origin, a wh-phrase generally moves overtly to a clause-initial position in wh-interrogative sentences. As is well known, such a movement cannot cross islands, namely, the structures out of which a constituent cannot be extracted. One of the famous islands is the complex NP island, where the complex NP refers to the NP modified by a clause (Bošković, 2016). The complex NP island/constraint requires that extraction from a complex NP is not allowed (Ross, 1967). For example, the clauses ‘that wrote’ and ‘that John wrote’ modify the NP ‘the book’ in (1a) and (1b), respectively. They are islands. After *who* in (1a) moves out of the relative clause ‘that wrote,’ the complex NP island/constraint will be violated, and the derived sentence will be ungrammatical. The same is true of (1b).

**Table:** 

(1)	a. *Who_*i*_ do you like the book_*j*_ that t_*i*_ wrote t_*j*_?
	b. *Why_*i*_ do you like the book_*j*_ that John wrote t_*j*_ t_*i*_?

However, in Mandarin Chinese (hereafter ‘Chinese’) and other East Asian languages, wh-elements stay in situ. Furthermore, different from adjunct *wh’s*-in-situ, argument *wh’s*-in-situ do not seem to be subject to island constraints, as (2) shows.

**Table:** 

(2)	a.	Nǐ	xǐhuan	shéi	xiě	de	shū?
		You	like	who	write	Rel	book.
	‘Who is the person x such that you like the book that (he/she) wrote?’						
	b.	*Nǐ	xǐhuan	Zhāngsān	wèishénme	xiě	de
		You	like	Zhangsan	why	write	Rel
		shū?					
		book.					
	‘What is the reason x such that you like the book [that Zhangsan wrote for x]?’						

This difference in island sensitivity between argument and adjunct *wh’s*-in-situ is known as argument–adjunct asymmetry in the literature.^1^ This phenomenon has drawn the interest of many scholars, and many influential hypotheses have been advanced to account for it (see Huang, 1982a,b; Lasnik and Saito, 1992; Aoun and Li, 1993a; Tsai, 1999; Cheng, 2009, among many others).

Inspired by the studies of Sprouse (2007), Sprouse et al. (2012), Sprouse and Hornstein (2013), Lu et al. (2020) used the acceptability judgment paradigm to investigate *wh’s*-in-situ in Chinese. They report that both argument *wh’s*-in-situ and adjunct ones are sensitive to the Complex NP Island. This study is interesting because if it is proved to be true, we would need to reconsider the existing theory of *wh’s*-in-situ that has been established based on the argument–adjunct asymmetry for *wh’s*-in-situ. Nevertheless, in this study we will point out that Lu et al.’s (2020) experimental design has some drawbacks, which make their findings unreliable. We critically note that when object *wh’s*-in-situ like *shénme* ‘what’ are located inside a relative clause, they are subject to a pragmatic constraint, suggesting that the VP (formed by a verb and its wh-object) in the relative clause tends to describe the prominent/salient feature of the relativized nominal head. Improving on the design in experimental materials, we conducted two experiments on Chinese *wh’s*-in-situ. The results of the experiments show that unlike adjunct *wh’s*-in-situ, argument *wh’s*-in-situ are not sensitive to island constraints, which is in line with the long-established findings on the issue at stake.

## The logic of factorial design for isolating the island effects and the previous formal judgment study into *wh*-in-situ

Let us first expound the logic of the factorial design for isolating island effects, on which the previous formal judgment study into *wh*-in-situ, namely Lu et al. (2020) is based. Sprouse and his colleagues argue that the lower acceptability of island violating sentences results not only from the violation of a grammatical constraint, but also from such (non-grammatical) processing factors as dependency length and structure. In other words, the processing of dependency length and complex structure also contributes to the low acceptability of island violating sentences. With the dependency length in a sentence becoming longer, the processing load of the sentence will increase and its acceptability will decrease. Likewise, the complex structure (i.e., the structure containing island) is more difficult to process than the simple one (i.e., the structure without an island). Given this, Sprouse and his colleagues developed a factorial experiment design to isolate island effects, which has been fruitfully adopted by many researchers to study island effects in various languages (Sprouse, 2007; Sprouse et al., 2011, 2012, 2016; Almeida, 2014; Michel, 2014; Atkinson et al., 2016; Kush et al., 2018, 2019; Stepanov et al., 2018; Keshev and Meltzer-Asscher, 2019; Pañeda et al., 2020; Kush and Dahl, 2022). The first factor in such an experiment paradigm is Dependency Length, which has two levels: long and short. In the short condition, a wh-phrase moves from a position in the matrix clause to the sentence-initial position, and in the long condition a wh-phrase moves from a position within the embedded clause to the sentence-initial position. The second factor is Structure, which also has two levels: island and non-island. The island condition contains an island, and the non-island condition does not. After the two levels of each factor are crossed, four conditions are created, as is demonstrated with a complex NP island below.

**Table:** 

(3)	a. Who __ heard that Jeff baked a pie? (Non-island + short)
	b. What did you hear that Jeff baked __? (Non-island + long)
	c. Who __ heard [the statement that Jeff baked a pie]? (Island + short)
	d. What did you [hear the statement that Jeff baked __]? (Island + long)
	(Sprouse et al., 2016, p. 318)

The island effect can be isolated with the logic of subtraction. First, the total effect is quantified by calculating the difference between (3a) and (3d) (i.e., [3a − 3d]), and then the effect of dependency length can be captured by calculating the difference between (3a) and (3b) (i.e., [3a − 3b]), and the effect of structure can be isolated by calculating the difference between (3a) and (3c) (i.e., [3a − 3c]). The island effect can now be obtained by subtracting the effect of dependency length and that of structure from the total effect. Put differently, the island effect can be quantified with the following formula: island effect = [3a − 3d] − [3a − 3b] − [3a − 3c]. If there is no island effect, the score for the island effect will be zero (in mathematic terms, [3a − 3d] = [3a − 3b] + [3a − 3c]). By contrast, if there is an island effect, the score for the island effect will be larger than zero.^2^ In other words, if the island effect is present, the total effect will be greater than the sum of the dependency length effect and the structure effect (in mathematic terms, [3a − 3d] > [3a − 3b] + [3a − 3c]), and the island effect is reflected by the super-additive effect. The island/super-additive effect can be identified statistically. If the island effect is present, there will be a significant interaction effect between the two factors: Dependency Length and Structure.

The formula for the island effect also has an equivalence like ‘island effect = [3d − 3c] − [3b − 3a].’ The formula can be interpreted as follows. [3b − 3a] can capture the effect of dependency length. If there is an island/super-additive effect, the difference between (3c) and (3d) should be greater than the difference between (3a) and (3b) though (3c) and (3d) appear to be different only by dependency length (Keshev and Meltzer-Asscher, 2019).

Inspired by the experimental paradigm in studying island effects, Lu et al. (2020) adopted a 2 × 2 × 2 factorial experiment design, involving the three factors such as *Dependency Length* (short vs. long), *Structure* (non-island vs. island) and *Wh-Category* (argument vs. adjunct). This gave the following eight conditions: (i) short + non-island + argument, (ii) long + non-island + argument, (iii) short + island + argument, (iv) long + island + argument, (v) short + non-island + adjunct, (vi) long + non-island + adjunct, (vii) short + island + adjunct, (viii) long + island + adjunct.

Consider the following sentences cited from their experiment, representing one set of their stimuli.

**Table:** 

(4)	Yuēhàn	xiǎngzhīdào	shéi	shuō	nǚhái
	John	wonders	who	say	girl
	chī-le	shòusī. (sh + nonisl + arg)			
	eat-Asp	sushi.			
	‘John wonders who said that the girl ate sushi.’				

**Table:** 

(5)	Yuēhàn	xiǎngzhīdào	bǐěr	shuō	nǚhái
	John	wonders	Bill	say	girl
	chī-le	shénme. (lo + nonisl + arg)			
	eat-Asp	what.			
	‘John wonders what Bill said that the girl ate.’				

**Table:** 

(6)	Yuēhàn	xiǎngzhīdào	shéi	jiàn-le	chī	shòusī
	John	wonder	who	meet-Asp	eat	sushi
	de	nǚhái. (sh + isl + arg)				
	Rel	girl.				
	‘John wonders who met the girl that ate sushi.’					

**Table:** 

(7)	Yuēhàn	xiǎngzhīdào	bǐěr	jiàn-le	chī	shénme
	John	wonder	Bill	meet-Asp	eat	what
	de	nǚhái. (lo + isl + arg)				
	Rel	girl.				
	‘John wonders what Bill met the girl that ate.’					

**Table:** 

(8)	Yuēhàn	xiǎngzhīdào	bǐěr	wèishénme	shuō	nǚhái
	John	wonders	Bill	why	say	girl
	chī-le	shòusī. (sh + nonisl + adj)				
	eat-Asp	sushi.				
	‘John wonders why Bill says that the girl ate sushi.’					

**Table:** 

(9)	Yuēhàn	xiǎngzhīdào	bǐěr	shuō	nǚhái	wèishénme
	John	wonders	Bill	say	girl	why
	chī-le	shòusī. (lo + nonisl + adj)				
	eat-Asp	sushi.				
	‘John wonders why Bill says that the girl ate sushi t.’					

**Table:** 

(10)	Yuēhàn	xiǎngzhīdào	bǐěr	wèishénme	jiàn-le	chī
	John	wonder	Bill	why	meet-Asp	eat
	shòusī	de	nǚhái. (sh + isl + adj)			
	sushi	Rel	girl.			
	‘John wonders why Bill met the girl that ate sushi.’					

**Table:** 

(11)	Yuēhàn	xiǎngzhīdào	bǐěr	jiàn-le	wèishénme	chī
	John	wonder	Bill	meet-Asp	why	eat
	shòusī	de	nǚhái. (lo + isl + adj)			
	sushi	Rel	girl.			
	‘John wonders why Bill met the girl that ate sushi.’ (Lu et al., 2020, p. 615)					

In these sentences, the verb *xiǎngzhīdào* ‘wonder’ takes an interrogative clause as its complement. For the sake of exposition, we will name the complement of *xiǎngzhīdào* ‘wonder’ as CP_1_. In the short condition, SpecCP_1_/C_1_ and the wh-phrase form mono-clausal dependency. If the wh-phrase is to move covertly to SpecCP_1_, such a movement in (4), (6), (8), and (10) will be a short-distance one. By contrast, in the long condition, SpecCP_1_/C_1_ and the wh-phrase form bi-clausal dependency. If the wh-phrase is to move covertly to SpecCP_1_, such a movement in (5), (7), (9), and (11) will be a long-distance one. The factor *Structure* controls for whether the complement of *xiǎngzhīdào* ‘wonder’ contains an island (the island condition) or not (the non-island condition). The factor *Wh-Category* controls for whether the wh-phrase at hand serves the role of an argument or an adjunct.

There were 24 target items and 72 filler sentences for the experiment. The participants were instructed to rate the naturalness of sentences on a seven-point Likert scale, with 1 being completely unnatural and 7 being completely natural. Their findings show that for both argument and adjunct *wh’s*-in-situ, there was a significant super-additive interaction effect of *Dependency Length* × *Structure*. This is an intriguing result because if proved to be true, their findings pose a challenge to the long-standing generalization on argument–adjunct asymmetry in *wh’s*-in-situ. That is, argument wh’s in Chinese are not subject to island constraints, whereas adjunct wh’s are (see, for example, Tsai, 1999). However, a careful inspection of Lu et al.’s (2020) experiment design indicates that we cannot be highly positive about the significance of their experiment because there are some drawbacks with their study. First, argument *wh’s*-in-situ can occur in both subject and object positions. In their design, they used two argument *wh’s*-in-situ, namely *shéi* ‘who’ and *shénme* ‘what.’ *Shéi* ‘who’ occurred in the short condition, serving as a subject, while *shénme* ‘what’ occurred in the long condition, serving as an object. But only *shénme* ‘what’ was manipulated to occur inside an island (i.e., only the island effects on the object *shénme* ‘what’ was tested). With the Complex NP Island as a test case, they argued that both argument and adjunct wh’s are subject to island constraints. This conclusion was too hastily drawn because the subject *shéi* ‘who’ was placed outside an island in their design (i.e., the island effects on the subject *shéi* ‘who’ were not tested),^3^ and there is no compelling evidence that object *wh’s*-in-situ are a typical case for testing island sensitivity. Without a detailed study of island effects on subject *wh’s*-in-situ, it would be particularly inappropriate to jump to the conclusion that both wh-argument and wh-adjunct elements are sensitive to island constraints. Second, when the object *wh*-in-situ in the relative clause is *shénme* ‘what,’ the interpretation of the construction is susceptible to factors not bearing on island constraints. One of the factors is that when object *wh’s*-in-situ are in a relative clause, the construction is subject to a pragmatic constraint, suggesting that the VP (formed by a verb and its wh-object) in the relative clause tends to characterize the prominent feature of the relativized nominal head.^4^ To illustrate the point, consider (7). Its alleged low acceptability might be caused by pragmatic inappropriateness rather than by a violation of an island constraint. To be exact, (7) is taken to be unacceptable because—without a proper context—eating a certain thing is not the prominent feature of a girl. On the other hand, if we provide an appropriate context, (7) will become acceptable. Suppose that there are three girls in a street eating different kinds of things: One girl is eating a hamburger, another girl, an omelet, and the third girl, fried chicken. If both the speaker and the hearer share this common ground, (7) will be acceptable because, in this context, eating a certain thing is the characteristic feature of the girls at issue, and it is natural to ask what the girl that Bill met ate.

The argument to the effect of displaying the role of a pragmatic factor in the interpretation of an island-internal object *wh*-in-situ is that when we replace the lexical item in (7) with other appropriate ones, the resulting sentences such as (12)–(14) will become acceptable too.

**Table:** 

(12)	Yuēhàn	xiǎngzhīdào	bǐěr	qǔ-le	yī-gè
	John	wonder	Bill	marry-Asp	one-Cl
	xǐhuan	chī	shénme	de	nǚhái.
	like	eat	what	Rel	girl.
	‘John wonders what is the thing x such that Bill married the girl who likes eating x.’				

**Table:** 

(13)	Yuēhàn	xiǎngzhīdào	bǐěr	mǎi-le	yī-běn	guānyú
	John	wonder	Bill	buy-Asp	one-Cl	about
	shénme	de	shū.			
	what	Rel	book.			
	‘John wonders what is the thing x such that Bill bought a book that is about x.’					

**Table:** 

(14)	Yuēhàn	xiǎngzhīdào	bǐěr	yùdào-le	jiāo
	John	wonder	Bill	meet-Asp	teach
	shénme	de	lǎoshī.		
	what	Rel	teacher.		
	‘John wonders what is the thing x such that Bill met the teacher that taught x.’				

For example, in the context of (12), the girl’s eating habit is important because after she and Bill got married, this would affect their relationship. Perhaps it will also affect John if he knows Bill. On this condition, it is natural to ask what the girl likes eating. The same kind of construal applies to (13). The content is a prominent feature of a book because in most cases whether the book can attract a person or not is dependent on its content. Consequently, it is valid to ask what the book that Bill bought is about. Likewise, teaching is the prominent feature of a teacher as the primary duty of a teacher is to teach. Given this, it is natural to ask what he taught, as in (14). However, Lu et al. (2020) fail to put the above pragmatic confounding factor under control.

To sum up, although Lu et al.’s (2020) results are intriguing, their experiment design still has at least two drawbacks: one is that they fail to study the island effects of subject *wh’s*-in-situ, and the other is that when studying the island effects of object *wh’s*-in-situ, they fail to put the pragmatic confounding factor under control. These drawbacks undermine their conclusions that argument *wh’s*-in-situ in Chinese are subject to an island constraint, and that there is no argument–adjunct asymmetry in *wh’s*-in-situ. In the next section, we will introduce our two experiments, in which the weaknesses of Lu et al. (2020) were resolved. In one experiment, the island sensitivity of subject *wh’s*-in-situ was tested, and in the other one, the island sensitivity of object *wh’s*-in-situ was tested with the pragmatic confounding factor under control.

## Our experiments

### Experiment 1: Island sensitivity of subject *wh’s*-in-situ

#### Participants

Ninety-six participants were recruited from a university in China, and each of them was paid 15 Yuan for taking part in the experiment.

#### Materials and methods

Correcting the potential problems with Lu et al.’s (2020) experimental design, we conducted an analogous experiment on the effects of the Complex NP island on *wh’s*-in-situ. Just as in Lu et al. (2020), the current experiment used a 2 × 2 × 2 factorial design, based on the following three factors: *Dependency Length* (short vs. long), *Structure* (non-island vs. island), and *Wh-Category* (argument vs. adjunct). Hence, this yielded eight conditions. The following examples are one set of the eight conditions constructed.

**Table:** 

(15)	Zhāngtāo	xiǎngzhīdào	shéi	juéde	Zhūlíng	huì
	Zhangtao	wonder	who	think	Zhuling	will
	mǎi	píngguǒ. (sh + nonisl + arg)				
	buy	apple.				
	‘Zhangtao wonders who thinks that Zhuling will buy apples.’					

**Table:** 

(16)	Zhāngtāo	xiǎngzhīdào	Zhūlíng	juéde	shéi	huì	mǎi
	Zhangtao	wonder	Zhuling	think	who	will	buy
	píngguǒ.(lo + nonisl + arg)						
	apple.						
	‘Zhangtao wonders who Zhuling thinks will buy apples.’						

**Table:** 

(17)	Zhāngtāo	xiǎngzhīdào	shéi	huì	chī	Zhūlíng	mǎi	de
	Zhangtao	wonder	who	will	eat	Zhuling	buy	Rel
	píngguǒ. (sh + isl + arg)							
	apple.							
	‘Zhangtao wonders who will eat the apples that Zhuling bought.’							

**Table:** 

(18)	Zhāngtāo	xiǎngzhīdào	Zhūlíng	huì	chī	shéi	mǎi	de
	Zhangtao	wonder	Zhuling	will	eat	who	buy	Rel
	píngguǒ.(lo + isl + arg)							
	apple.							
	‘Zhangtao wonders who is the person x such that Zhuling will eat the apples that x bought.’							

**Table:** 

(19)	Zhāngtāo	xiǎngzhīdào	ni	wèishénme	juéde	Zhūlíng
	Zhangtao	wonder	you	why	think	Zhuling
	huì	mǎi	píngguǒ. (sh + nonisl + adj)			
	will	buy	apple.			
	‘Zhangtao wonders why you think that Zhuling will buy apples.’					

**Table:** 

(20)	Zhāngtāo	xiǎngzhīdào	ni	juéde	Zhūlíng	wèishénme
	Zhangtao	wonder	you	think	Zhuling	why
	huì	mǎi	píngguǒ. (lo + nonisl + adj)			
	will	buy	apple.			
	‘Zhangtao wonders what is the reason x such that you think Zhuling will buy apples for x.’					

**Table:** 

(21)	Zhāngtāo	xiǎngzhīdào	ni	wèishénme	huì	chī	Zhūlíng
	Zhangtao	wonder	you	why	will	eat	Zhuling
	mǎi	de	píngguǒ. (sh + isl + adj)				
	buy	Rel	apple.				
	‘Zhangtao wonders why_*i*_ you t_*i*_ will eat the apples that Zhuling bought.’						

**Table:** 

(22)	Zhāngtāo	xiǎngzhīdào	ni	huì	chī	Zhūlíng
	Zhangtao	wonder	you	will	eat	Zhuling
	wèishénme	mǎi	de	píngguǒ. (lo + isl + adj)		
	why	buy	Rel	apple.		
	‘Zhangtao wonders what is the reason x such that you will eat the apples [that Zhuling bought for x].’					

In total, there were 24 target items in this experiment, and each item consisted of the eight conditions listed above. We thus had 192 target sentences. Using Latin Square, we assigned all of these sentences to eight lists. Consequently, each list had 24 test/target sentences and 72 filler sentences. With four practice sentences, each list had 100 sentences in total. Each list was pseudo-randomized, so that the sentences of the same experimental condition would not be adjacent. After that, each list was counterbalanced into four orders in order to remove the confounding factor of order.

Just like Lu et al. (2020), our test was administered with paper questionnaires. The participants were instructed to rate the naturalness of the sentences on a seven-point Likert scale, with 1 being completely unnatural and 7 being completely natural. As can be seen, our design was similar to that of Lu et al. (2020). Nevertheless, there were some crucial differences between our experiment and theirs. In our experiment we only employed *shéi* ‘who’ for the argument *wh’s*-in-situ. In the condition of short dependency, *shéi* ‘who’ served as the subject of the first embedded clause, and in the condition of long dependency, *shéi* ‘who’ served either as the subject of the second embedded clause or as that of the relative clause/CNP. In other words, the grammatical roles of argument *wh’s*-in-situ were held constant across conditions, which helped to exclude the confounding factors such as thematic role and word order. In addition, we carefully selected the words to ensure that in all the conditions, wh-phrases in long dependency were placed two words/four syllables further away from *xiǎngzhīdào* ‘wonder’ than those in short dependency, which ensured that the effect of *Dependency Length* could be subtracted from the results.

Since *wèishénme* ‘why’ is ambiguous, the participants were told at the very beginning of the experiment that *wèishénme* ‘why’ in all the experimental sentences was to question the reason of an action/event, which is similar to the English counterpart *why* rather than *for what*.^5^ This was intended to prevent an unnecessary processing load caused by ambiguity resolution. To check whether the participants correctly understood the instructions, we asked them to provide answers to the experiment items. To be exact, they were told that xiǎngzhīdáo ‘wonder’ takes an interrogative clause as its complement. They were asked to provide an answer to the interrogative clause containing *wèishénme*. Their answers were supposed to be based on their interpretation of the wh-phrase in making the acceptability judgment—that is, whether under such an interpretation the relevant interrogative clause is unacceptable or not. If the participant’s answer to the question containing *wèishénme* is *yīnwèi*… ‘because…’ or *yóuyú*… ‘since…,’ etc, it would indicate that he/she understood the instructions correctly. By contrast, if his/her answer to *wèishénme* is *wèile* … ‘for the purpose of …,’ all his/her scores would be excluded from the data because he/she would have failed to understand our instructions.

As noted by Tsai (2008), when interpreted as ‘why,’ *wèishénme* can only be used in front of the future modal *huì*. If preceded by the future modal *huì* as in (23a), it cannot be interpreted as ‘why.’ However, if in this context it is interpreted as ‘for what,’ as *wèile shénme* is in (23b), then the sentence will be acceptable. Based on Tsai (2008), we added some filler items like (23). If a participant’s mean acceptability score for fillers like (23a) is higher than 5, then all his/her rating scores would be removed from the resulting data even if his/her answer to *wèishénme* is *yīnwèi*… ‘because…’ or *yóuyú*… ‘since….’ The reason is that giving such a high score might indicate that the participant unconsciously interpreted *wèishénme* as ‘for what,’ defiant of our instruction.

**Table:** 

(23)	a.	*Zhāngsān	xiǎngzhīdào	Lǐsí	huì	
		Zhangsan	wonder	Lisi	will	
		wèishénme	cídiào	nàfèn	gōngzuò.	
		why	quit	that	job.	
		‘Zhangsan wonders why Lisi will quit that job.’				
	b.	Zhāngsān	xiǎngzhīdào	Lǐsí	huì	wèile
		Zhangsan	wonder	Lisi	will	for
		shénme	cídiào	nàfèn	gōngzuò.	
		what	quit	that	job.	
		‘Zhangsan wonders for what Lisi will quit that job.’				

#### Predictions

If Lu et al. (2020) are on the right track, we expect that there should be no difference between argument and adjunct *wh’s*-in-situ in terms of island effects. Put differently, there should be a super-additive interaction, or a super-additive effect between *Structure* and *Dependency Length* factors for both argument and adjunct *wh’s*-in-situ. If this is true, we should also expect no three-way interaction of *Structure* × *Wh-Category* × *Length*. On the contrary, provided that argument and adjunct *wh’s*-in-situ are different in terms of island effects, we should expect to find a significant three-way interaction in *Structure* × *Wh-Category* × *Length.*

#### Results

We found that *wèishénme* was correctly interpreted by all the participants. Then, following Sprouse et al. (2012) and Kush et al. (2018), among others, we built a regression model to analyze the acceptability rating scores. Using the Lme4 package in R (R Core Team, 2021), we constructed linear mixed-effects models, with Structure, Dependency Length, WH-category, and their interactions as the fixed effects. Each model was initially built with maximum random intercepts and random slopes for participants, and the random slope was eliminated stepwise if the model failed to converge. We calculated p-values for the main effects of Structure, Dependency length, WH-category, and their interactions using the lmerTest package (Kuznetsova et al., 2017).^6^ The results showed a significant main effect of *Structure* (β = 1.87, SE = 0.14, *t* = 13.69, *p* < 0.001), of *Length* (β = 4.34, SE = 0.11, *t* = 40.01, *p* < 0.001), and of *Wh-Category* (β = 3.94, SE = 0.12, *t* = 31.88, *p* < 0.001). Contrary to the findings of Lu et al. (2020), there were significant effects of two-way interactions such as *Structure* × *Length* (β = −2.46, SE = 0.15, *t* = −16.02, *p* < 0.001), *Structure* × *Wh-Category* (β = −3.26, SE = 0.17, *t* = −19.23, *p* < 0.001), and *Length* × *Wh-Category* (β = −3.91, SE = 0.15, *t* = −25.52, *p* < 0.001). Furthermore, we did find that there was a significant interaction in *Structure* × *Wh-Category* × *Length* (β = 2.23, SE = 0.22, *t* = 10.29, *p* < 0.001). See also the Supplementary material for the complete model results.

**FIGURE 1:**
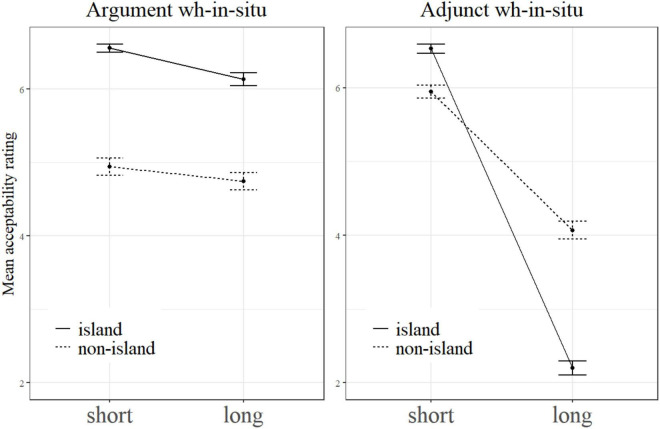
The interaction plots for argument *wh*-in-situ and adjunct *wh*-in-situ.

On a par with Lu et al. (2020), we also put *Structure* × *Length* under the levels of *Wh-Category* to examine the island sensitivity effect of each wh-category. The result showed that as for the wh-adjunct *wèishénme*, there was a significant super-additive interaction effect between *Structure* and *Dependency Length* (β = −2.46, SE = 0.19, *t* = −12.81, *p* < 0.001). As for the wh-argument *shéi* in subject position, however, no super-additive effect was observed (β = −0.23, SE = 0.15, *t* = −1.52, *p* = 0.13). In other words, the wh-argument and the wh-adjunct exhibited a significant difference regarding island effects. This can be seen clearly in Figure 1 where the left panel shows the interaction plot for argument *wh*-in-situ and the right panel presents the interaction plot for adjunct *wh*-in-situ.

**FIGURE 2:**
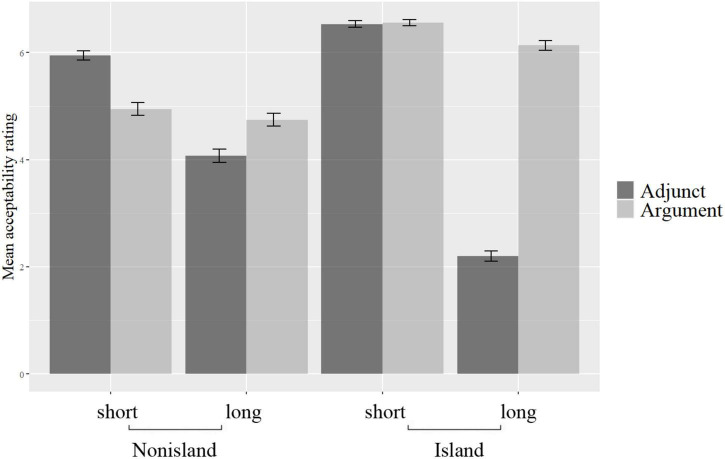
Mean acceptability rating scores.

The mean acceptability scores are presented in Figure 2, with the error bars representing the standard errors.

As can be seen from Figure 2, it is not the case that the argument *wh*-in-situ is generally rated to be better than the adjunct *wh*-in-situ, contrary to the findings of Lu et al. (2020). In the short + island condition, the argument and the adjunct *wh’s*-in-situ exhibit no significant difference in acceptability. Notably, in the short + non-island condition, the adjunct *wh*-in-situ is better than the argument *wh*-in-situ. However, it is in the long + island condition that the adjunct *wh*-in-situ is significantly worse than the argument *wh*-in-situ, with their mean rating scores being 2.20 and 6.14, respectively.

### Experiment 2: Island sensitivity of object *wh’s*-in-situ

It is pointed out in Section “The logic of factorial design for isolating the island effects and the previous formal judgment study into *wh*-in-situ” that when the object *wh*-in-situ *shénme* ‘what’ is in a relative clause island, its interpretation is susceptible to factors not bearing on the Complex NP Constraint. Specifically, the object *wh*-in-situ in the relative clause is subject to a pragmatic constraint, such that the VP (formed by a verb and the following wh-object) in the relative clause is bound to characterize the prominent feature of the relativized nominal head. In this experiment, putting this pragmatic confounding factor under control, we intend to ascertain whether there is argument-adjunct asymmetry in island sensitivity of *wh’s*-in-situ.

#### Participants

Another group of 64 participants were recruited from a university in China,^7^ and each of them was paid 15 Yuan for taking part in Experiment 2.

#### Materials and methods

Just like Lu et al.’s (2020) experimental design, the current experiment also employed a 2 × 2 × 2 factorial design, based on the following three factors: *Dependency Length* (short vs. long), *Structure* (non-island vs. island), and *Wh-Category* (argument vs. adjunct). Therefore, this yielded the eight conditions. Similar to Lu et al.’s (2020) experimental design, but different from our Experiment 1, the *wh*-in-situ in the short condition is served by *shéi* ‘who,’ and the one in the long condition is served by *shénme* ‘what.’ The following examples are one representative set of the eight conditions constructed.

**Table:** 

(24)	Zhōuyǒng	xiǎngzhīdào	shéi	juéde	lièrén	huì
	Zhouyong	wonder	who	think	hunter	will
	bǔshā	jīngyú. (sh + nonisl + arg)				
	kill	whale				
	‘Zhouyong wonders who thinks that the hunter will kill whales.’					

**Table:** 

(25)	Zhōuyǒng	xiǎngzhīdào	zhèngfǔ	juéde
	Zhouyong	wonder	government	think
	lièrén	huì	bǔshā	
	hunter	will	kill	
	shénme. (lo + nonisl + arg)			
	what			
	‘Zhouyong wonders what the government thinks that the hunter will kill.’			

**Table:** 

(26)	Zhōuyǒng	xiǎngzhīdào	shéi	huì	chéngfá
	Zhouyong	wonder	who	will	punish
	bǔshā	jīngyú	de	lièrén.	(sh + isl + arg)
	kill	whale	Rel	hunter	
	‘Zhouyong wonders who will punish the hunter that kills whales.’				

**Table:** 

(27)	Zhōuyǒng	xiǎngzhīdào	zhèngfǔ	huì
	Zhouyong	wonder	government	will
	chéngfá	bǔshā	shénme	de
	punish	kill	what	Rel
	lièrén. (lo + isl + arg)			
	hunter			
	‘Zhouyong wonders what is the thing x such that the government will punish the hunter who kills x.’			

**Table:** 

(28)	Zhōuyǒng	xiǎngzhīdào	zhèngfǔ	wèishénme
	Zhouyong	wonder	government	why
	juéde	lièrén	huì	bǔshā
	think	hunter	will	kill
	jīngyú. (sh + nonisl + adj)			
	whale			
	‘Zhouyong wonders why the government thinks that the hunter will kill whales.’			

**Table:** 

(29)	Zhōuyǒng	xiǎngzhīdào	zhèngfǔ	juéde	lièrén
	Zhouyong	wonder	government	think	hunter
	wèishénme	huì	bǔshā	jīngyú. (lo + non-is + adj)	
	why	will	kill	whale	
	‘Zhouyong wonders what is the reason x such that the government thinks that the hunter will kill whales for x.’				

**Table:** 

(30)	Zhōuyǒng	xiǎngzhīdào	zhèngfǔ	wèishénme
	Zhouyong	wonder	government	why
	huì	chéngfá	bǔshā	jīngyú
	will	punish	kill	whale
	de	lièrén. (sh + isl + adj)		
	Rel	hunter		
	‘Zhouyong wonders why the government will punish the hunter who kills whales.’			

**Table:** 

(31)	Zhōuyǒng	xiǎngzhīdào	zhèngfǔ	huì	chéngfá
	Zhouyong	wonder	government	will	punish
	wèishénme	bǔshā	jīngyú	de	
	why	kill	whale	Rel	
	lièrén. (lo + isl + adj)				
	hunter				
	‘Zhouyong wonders what is the reason x such that the government will punish the hunter [who kills whales for x].’				

Another set of three experimental sentences for the long + island + argument condition is shown below to demonstrate how the pragmatic confounding factor is controlled for.

**Table:** 

(32)	Hánbīn	xiǎngzhīdào	Yánliàng	huì	shōumǎi	jiǎnchá
	Hanbin	wonder	Yanliang	will	bribe	inspect
	shénme	de	jǐngchá.			
	what	Rel	policeman.			
	‘Hanbin wonders what is the thing x such that Yanliang will bribe the policeman who inspects x.’					

**Table:** 

(33)	Sūnhǎi	xiǎngzhīdào	Lǔxiáng	huì	zhāo	shàncháng
	Sunhai	wonder	Luxiang	will	recruit	be-good-at
	shànme	de	xiāoshòu.			
	what	Rel	salesperson.			
	‘Sunhai wonders what is the thing x such that Luxiang will recruit the salesperson who is good at x.’					

**Table:** 

(34)	Cáomíng	xiǎngzhīdào	Kǒngwén	huì	guānzhù
	Caoming	wonder	Kongwen	will	pay-attention
	bàodào	shénme	de	xīnwén.	
	report	what	Rel	news.	
	‘Caoming wonders what is the thing x such that Kongwen will pay attention to the news that reports x.’				

As can be seen in (32)–(34), all the verbs in the relative clauses are related to the prominent features of the relativized nominal heads. To illustrate this point, consider (32). The relativized nominal head *jǐngchá* ‘policeman’ has different kinds of features. He can engage in different kinds of actions. For example, a policeman can drink water, watch TV, read books, inspect something, etc. In the context of (32), the feature like ‘inspect something’ becomes prominent because after reading ‘Yánliàng will bribe the policeman,’ one expects to know something associated with this policeman’s duty. Put differently, the use of the verb *jiǎnchá* ‘inspect’ can render the whole sentence coherent. By contrast, if we change this verb to *chī* ‘eat’ or *hē* ‘drink,’ the acceptability of the sentence such as (35) will decrease substantially because ‘eat something’ and ‘drink something’ are not prominent actions or features associated with *jǐngchá* ‘policeman.’

**Table:** 

(35)	Hánbīn	xiǎngzhīdào	Yánliàng	huì	shōumǎi	hē
	Hanbin	wonder	Yanliang	will	bribe	drink
	shénme	de	jǐngchá.			
	what	Rel	policeman.			
	‘Hanbin wonders what is the thing x such that Yanliang will bribe the policeman who drinks x.’					

Consider also (33). When one wants to recruit a salesperson, he will pay attention to the person’s ability or specialty. Therefore, being good at something will be one of the prominent features of this person. The same is true of (27) and (34). The main activity associated with a hunter is to capture and kill something, and the main function of news is to report something. In other words, bǔshā ‘capture and kill’ and *bàodào* ‘report’ are used to describe the prominent features of the corresponding relativized nominal heads. For more discussion about the notion of prominence adopted in this study, see Ariel (2019) and references therein, among others.

Other designs of this experiment are similar to those of Experiment 1. For example, just like Experiment 1, we also used four lexicalizations of the sentence type in (23a) (repeated as (36)) as part of our filler items. If a participant’s mean acceptability score for sentences like (36) was more than 5, all his/her scores would be removed. For the sake of space and simplicity, we will not reiterate the introduction of the experiment design.

**Table:** 

(36)	*Zhāngsān	xiǎngzhīdào	Lǐsí	huì	wèishénme	cídiào
	Zhangsan	wonder	Lisi	will	why	quit
	nàfèn	gōngzuò.				
	that	job.				
	‘Zhangsan wonders why Lisi will quit that job.’					

#### Predictions

If Lu et al. (2020) are on the right line, we expect that there should be no difference between argument and adjunct *wh’s*-in-situ in terms of island effects. In other words, there should be a super-additive interaction, or a super-additive effect between *Structure* and *Dependency Length* factors for both argument and adjunct *wh’s*-in-situ. Along this line, we should also expect no three-way interaction of *Structure* × *Wh-Category* × *Length*. Unlike Lu et al. (2020), however, we argue that the low acceptability score of experimental items containing argument *wh’s*-in-situ in relative clause islands, reported by Lu et al. (2020), is suspected to stem from pragmatic inappropriateness due to the constructional idiosyncrasy of relative clauses. Put differently, the reported lack of argument-adjunct asymmetry in Lu et al. (2020) is taken to result from the confounding effect of a pragmatic constraint at work for object *wh’s*-in-situ. If our assessment of Lu et al. (2020) is correct, after fixing the experimental materials so that the pragmatic confounding effect is controlled for or eliminated, we expect that the result will be different from theirs in light of argument vs. adjunct asymmetry of island effects, and we should thus expect of our experiment a significant three-way interaction in *Structure* × *Wh-Category* × *Length*.

#### Results

The rating scores of two participants were removed from subsequent analysis because both rated all the sentences like (36) as 7, which indicates that they did not follow our instruction and interpreted *wèishénme* as ‘for what’ rather than ‘why.’^8^ Then, the acceptability rating scores were analyzed with same method as that of Experiment 1. The results showed significant main effects of *Structure* (β = 2.74, SE = 0.17, *t* = 16.03, *p* < 0.001), of *Length* (β = 4.58, SE = 0.13, *t* = 34.48, *p* < 0.001), and of *Wh-Category* (β = 4.05, SE = 0.14, *t* = 29.35, *p* < 0.001). Contrary to the findings of Lu et al. (2020), however, there were significant effects of two-way interactions such as *Structure* × *Length* (β = −2,99, SE = 0.17, *t* = −17.91, *p* < 0.001), *Structure* × *Wh-Category* (β = −3.01, SE = 0.18, *t* = −16.48, *p* < 0.001), and *Length* × *Wh-Category* (β = −4.17, SE = 0.17, *t* = −24.91, *p* < 0.001). Furthermore, there was a significant interaction in *Structure* × *Wh-Category* × *Length* (β = 2.74, SE = 0.24, *t* = 11.59, *p* < 0.001).

**FIGURE 3:**
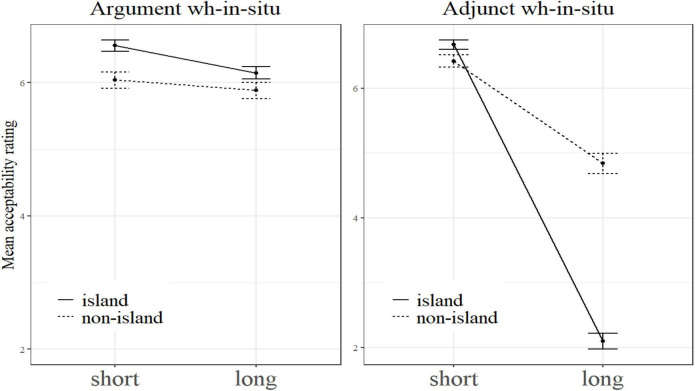
The interaction plots for argument *wh*-in-situ and adjunct *wh*-in-situ.

On a par with Lu et al. (2020) and Experiment 1, we also put *Structure* × *Length* under the level of *Wh-Category* to examine the island sensitivity effects of each wh-category. The result showed that as for the wh-adjunct *wèishénme* ‘why,’ there was a significant super-additive interaction effect between *Structure* and *Dependency Length* (β = −2.99, SE = 0.17, *t* = −17.99, *p* < 0.001). As for the wh-arguments, however, no super-additive effect was observed (β = −0.25, SE = 0.16, *t* = −1.62, *p* = 0.11). Put differently, the wh-arguments and the wh-adjuncts exhibited a significant difference in island effects. This can be seen clearly in Figure 3 where the left panel shows the interaction plot for argument *wh*-in-situ and the right panel presents the interaction plot for adjunct *wh*-in-situ.

**FIGURE 4:**
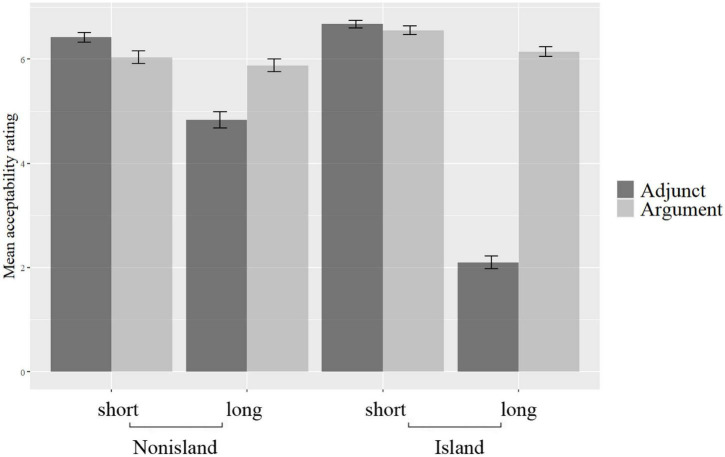
Mean acceptability rating scores.

The mean acceptability scores are presented in Figure 4, with the error bars representing the standard errors.

As can be observed, just like those of Experiment 1, the mean acceptability scores in Figure 4 exhibit different patterns from those of Lu et al. (2020). It is not the case that the argument *wh’s*-in-situ are generally rated to be better than the adjunct *wh*-in-situ. Notably, in the short + non-island condition the adjunct *wh*-in-situ is better than the argument *wh*-in-situ *shéi* ‘who.’ Moreover, in the short + island condition the adjunct *wh*-in-situ is slightly better than the argument *wh*-in-situ *shéi* ‘who.’ It is worth noting that in the long + island condition the adjunct *wh*-in-situ is significantly worse than the argument *wh*-in-situ *shénme* ‘what,’ with their mean rating scores being 2.10 and 6.15, respectively.

## General discussion

This study brings to light several important findings. First, a significant three-way interaction effect of *Structure* × *Wh-Category* × *Dependency Length* was observed. In this regard, our study renders reinforcing support to the long-standing generalization concerning the argument–adjunct asymmetry in *wh’s*-in-situ in Chinese (Huang, 1982a,b; Tsai, 1999; Cheng, 2009), contrary to the findings of Lu et al. (2020). In accordance with Sprouse (2007), Sprouse and Hornstein (2013), and Sprouse et al. (2016), to name a few, syntactic island effects can be measured systematically using the factorial design, and they are represented by the super-additive effects that stem from combining both the effects of *Structure* and those of *Dependency Length*. Adopting this experimental paradigm, we have demonstrated that the wh-adjunct *wèishénme* in the current study displays island effects as there arises a significant *Structure* × *Dependency Length* interaction giving rise to the super-additive effect. This, in turn, supports the well-acknowledged claim that wh-adjuncts are sensitive to island constraints (Huang, 1982a,b), or that covert (operator) movement is involved in the derivation of wh-adjuncts (Aoun and Li, 1993a; Tsai, 1999). By contrast, in the current study no super-additive effect is observed for wh-arguments, which counters the findings of Lu et al. (2020). This means that the traditional generalization should be re-endorsed—that is, wh-arguments in Chinese are not sensitive to island constraints. The underlying reason behind this re-endorsement is that wh-arguments are derived by unselective binding (Tsai, 1994, 1999), or that an operator licensing them is base-generated in a position external to islands (Aoun and Li, 1993a). Put differently, the significant difference between the adjunct *wh’s*-in-situ and the argument *wh’s*-in-situ in the long + island condition reflects the different syntactic properties of the adjunct *wh’s*-in-situ and the argument *wh’s*-in-situ. The argument *wh’s*-in-situ do not undergo covert movement. Without violating the island constraint, the relevant sentences are rated to be natural. By contrast, the adjunct *wh’s*-in-situ undergo covert movement, violating the island constraint. Consequently, the relevant sentences are rated to be unnatural.^9^

Second, Lu et al.’s (2020) experiment result shows that although the mean acceptability rating score of the long + island + argument condition is low, it is still a little higher than that of the long + island + adjunct condition. Given this, they acknowledge that Huang (1982a,b) and Tsai (1994, 1999) are correct in noting that argument *wh’s*-in-situ in islands are higher in acceptability than adjunct *wh’s*-in-situ in islands. Nevertheless, they argue that it does not support the argument-adjunct asymmetry in that it may be a reflection of the main effect of *Wh-Category*. They further argue that there is no real argument–adjunct asymmetry in *wh’s*-in-situ in terms of island sensitivity. The argument-adjunct asymmetry in *wh’s*-in-situ reported in the literature is attributed to the methodology of acceptability judgment. In their opinion, while making judgments, linguists implicitly construct minimal pairs, i.e., they implicitly employ a minimal-pair experiment design. In the experiment employing such a design, the preference for wh-arguments in situ rather than wh-adjuncts in situ would be a reflection of the main effect of *Wh-Category* (argument vs. adjunct) rather than wh-adjuncts’ sensitivity to island constraints. Our two experiments have demonstrated that the validity of their claim needs to be re-evaluated. Recall the results of Experiment 1 and Experiment 2. They reveal that the wh-arguments were not always rated to be better in acceptability than the wh-adjunct, which is different from the findings made in Lu et al. (2020). Particularly, our two experiments have found that in the short + non-island condition, the wh-adjunct was judged to be more acceptable than the wh-arguments. Further, no significant difference between the wh-argument and the wh-adjunct was observed in the short + island condition of Experiment 1, and the wh-adjunct was slightly better than the wh-argument in the same condition of Experiment 2.^10^ Based on the findings in our study, we can conclude that even if Lu et al.’s (2020) assumption is reasonable that while making judgments, linguists implicitly employ a minimal-pair experiment design, the preference for argument *wh’s*-in-situ in the island condition cannot be attributed to the effects of *Wh-Category*.

In addition, it is worth noting that previous studies often focus only on island effects that arise on adjunct *wh’s*-in-situ, without explicit reference to minimal pairs involving both adjunct *wh’s*-in-situ and argument *wh’s*-in-situ. It is, then, implausible to claim that the participants in these studies always implicitly construct a minimal pair, which—as Lu et al. (2020) claim—leads to argument–adjunct asymmetry in island sensitivity.

Perhaps, at this moment one may wonder why the wh-adjunct in the short + non-island condition was judged to be more acceptable than the wh-argument in our experiments and why there was a difference in this regard from Lu et al. (2020). As to the first question, we think the answer might lie in different sensitivity to dependency length between argument *wh’s*-in-situ and adjunct *wh’s*-in-situ. As can be seen from the two interaction plots of our experiments, the dotted lines for argument *wh’s*-in-situ in the left panels are almost flat, and the dotted ones for adjunct *wh’s*-in-situ in the right panels slope downward visibly. The identical result is also found in Lu et al. (2020). This suggests that adjunct *wh’s*-in-situ are more sensitive to dependency length than argument *wh’s*-in-situ. Therefore, it is highly likely that the mean acceptability rating score for wh-adjuncts is higher than or approximately equal to that of wh-arguments (when the processing difference between adjunct *wh’s*-in-situ and argument *wh’s*-in-situ is small) in short dependency condition, and the rating score for wh-adjuncts is lower than that of wh-arguments in long dependency condition. Given this, it will be unsurprising that in the short + non-island condition, wh-adjuncts were judged to be more acceptable than wh-arguments.

If we continue to investigate why there is a difference in sensitivity to dependency length, we speculate that the reason might be that wh-adjuncts and wh-arguments establish dependency in different ways: wh-adjuncts establish dependency through LF movement and wh-arguments through unselective binding (Tsai, 1999). It will be a very interesting topic to study whether varying degrees of sensitivity to dependency length can be used as a criterion for determining how dependency is established. We leave this topic for future study.

As to the question why argument *wh’s*-in-situ are preferred over adjunct *wh’s*-in-situ in the short + non-island condition in Lu et al.’s experiment, a result different from ours, we speculate that one reason lies in the choice of an embedded subject. The embedded subject in the adjunct + short + non-island condition of their experiment is a proper noun, as in (8). It has been reported in the literature that a proper noun/definite phrase provokes a higher processing cost (Warrena and Gibson, 2002; Hofmeister and Sag, 2010), which will reduce the acceptability of this type of sentence. Therefore, it is reasonable that the mean rating score for argument *wh’s*-in-situ is greater than that of adjunct *wh’s*-in-situ in Lu et al.’s experiment. In contrast to the stimuli in Lu et al.’s experiment, the embedded subject in the adjunct + short + non-island condition of our experiment is a pronoun, as in (19). The embedded subject may thus not have induced any additional processing cost. This may have resulted in the explicit short dependency effect of adjunct *wh’s*-in-situ. Consequently, the mean rating score for adjunct *wh’s*-in-situ is greater than that of argument *wh’s*-in-situ.

Third, we suspect, as pointed above, that the low acceptability score reported by Lu et al. (2020) concerning wh-arguments in the long + island condition results from pragmatic inappropriateness of their experimental items rather than the island constraint at stake. In other words, we propose that when the object *wh*-in-situ is in a relative clause/an island, the processing of such a sentence is susceptible to an additional pragmatic constraint. This is confirmed by Experiment 2, in which the experiment items were carefully designed so that the pragmatic confounding factor could be removed. The results of this experiment have shown that once the pragmatic confounding factor is under control, the acceptability score for the object *wh*-in-situ in the long + island condition is very high.

Moreover, a comparison of the results of Experiment 1 and Experiment 2 also suggests that we should be on the right track in assuming that when the object *wh*-in-situ is in a relative clause/an island, it is subject to an additional pragmatic constraint, and the low acceptability score reported by Lu et al. (2020) concerning wh-arguments in the long + island condition results from pragmatic inappropriateness. The results of Experiment 1 and Experiment 2 have revealed that the subject *wh*-in-situ and the object *wh*-in-situ behave almost in the same way. For example, in the long + island condition, both the subject *wh*-in-situ and the object *wh*-in-situ are judged to be better than the adjunct *wh*-in-situ, and in the short + non-island condition, both the subject *wh*-in-situ and the object *wh*-in-situ are considered to be worse than the adjunct *wh*-in-situ. Their parallel behaviors are well expected because both subject and object *wh’s*-in-situ serve as arguments. Unlike Experiment 2, Lu et al. (2020) left the pragmatic confounding factor untouched. As a result, they reported that both the object *wh*-in-situ and the adjunct *wh*-in-situ in the long + island condition are rated to be very low in acceptability. If their findings are reasonable, then object *wh’s*-in-situ and adjunct *wh’s*-in-situ are distinguished in acceptability from the subject *wh*-in-situ in the long + island condition. Such a difference is surprising given that both subject *wh*-in-situ and object *wh*-in-situ are arguments. After fixing the experimental materials so that the pragmatic confounding effect is controlled for or eliminated, Experiment 2 has shown that the object *wh*-in-situ exhibits the same characteristics as the subject *wh*-in-situ does. This argues strongly in favor of our claim that it is the pragmatic confounding factor that prevents Lu et al. (2020) from uncovering the real nature of object *wh’s*-in-situ. Put differently, under the impact of the pragmatic confounding factor, their result shows that just like adjunct *wh’s*-in-situ, there is also a significant *Structure* × *Dependency Length* interaction for argument *wh’s*-in-situ. Consequently, they fail to find a significant three-way interaction of Structure × Wh-Category × Length. Actually, the significant *Structure* × *Dependency Length* interaction for argument *wh’s*-in-situ in Lu et al. (2020) is a reflection of pragmatic constraint rather than syntactic island constraint.

Our claim can also explain the practice that theoretical linguists in Chinese generally rely on: when intending to show that argument *wh’s*-in-situ in this language are not sensitive to island constraints, they usually use subject *wh’s*-in-situ as in (37) rather than object *wh’s*-in-situ as a test case.^11^

**Table:** 

(37)	Nǐ	xǐhuan	shéi	xiě	de	shū?
	You	like	who	write	Rel	book.
	‘Who is the person x such that you like the book that (he/she) wrote?’					

According to the findings of our experiments, the reason lies in that unlike subject *wh’s*-in-situ, when object *wh’s*-in-situ occur in a relative clause, namely an island, they are more likely subject to an additional pragmatic constraint. Therefore, it is much easier to create sentences with appropriate subject *wh’s*-in-situ than sentences with object *wh’s*-in-situ to examine the island sensitivity of argument *wh’s*-in-situ.

## Conclusion

Different from their counterparts in English, wh-elements in Chinese remain in situ in question sentences. Argument *wh’s*-in-situ in Chinese are insensitive to island constraints, unlike adjunct *wh’s*-in-situ. This has led to the celebrated generalization regarding the argument and adjunct asymmetry in *wh’s*-in-situ in this language. With the acceptability judgment experiment, Lu et al. (2020) challenge this long-established generalization. They argue that this asymmetry is an illusion, and that both the adjunct *wh*-in-situ and the argument *wh’s*-in-situ are subject to island constraints. In this study, we point out that their results are not convincing because their experimental design has some drawbacks. We redesigned the experimental materials in question to examine island effects on wh-elements in situ in Chinese. The results of the two experiments in this study show that the argument versus adjunct asymmetry in *wh’s*-in-situ is present. Furthermore, the argument–adjunct asymmetry at issue cannot be attributed to the main effect of *Wh-Category*.

On top of supporting the traditional theoretical generalization on Chinese *wh’s*-in-situ, this study also discovers that when object *wh’s*-in-situ are located inside a relative clause, they are subject to a pragmatic constraint, suggesting that the verb phrase in the relative clause tends to describe the prominent/salient feature of the relativized nominal head. This contributes to the understanding of the processing of relative clauses and *wh*-in-situ sentences.

Finally, it will be much fair to point out that although Lu et al.’s (2020) findings are not supported by our experiments, their research is very enlightening. Inspired by their study, in the future we will examine whether the argument-adjunct asymmetry is also observed in other island environments and whether our findings in this study may shed some light on other theories related to asymmetry in unselecting binding, covert LF movement, ECP, etc.

## Data availability statement

The raw data supporting the conclusions of this article will be made available by the authors, without undue reservation.

## Author contributions

QT conceived and designed the study, implemented the experiments, analyzed the data, drafted, and revised the manuscript. M-KP provided theoretical guidance when necessary and made revisions to the manuscript. XY participated in the conception of the study, statistical analysis, as well as the revision of part of the manuscript. All authors contributed to the article and approved the submitted version.

## Footnotes

1 Actually, the description “argument vs. adjunct asymmetry in *wh’s*-in-situ” is inaccurate. The more accurate description is “nominal and non-nominal asymmetry in *wh’s*-in-situ” (Tsai, 1999, 2008). For the sake of coherence, in this study we follow Lu et al. (2020) in using argument vs. adjunct asymmetry in *wh’s*-in-situ.

2 The number size can be used to measure the size of island effects. The focus of this study is to see whether island effects can be detected with *wh’s*-in-situ rather than to see the size of island effects. Given that the *p*-values for the interaction effects of the two experimental factors and the interaction plots are enough to show whether island effects are present, for the sake of simplicity we will follow Lu et al. (2020) in only reporting *p*-values and the interaction plots.

3 We do not suggest that test sentences should be multiple questions, containing both subject and object *wh’s*-in-situ or that subject *shéi* ‘who’ should be placed in an island in the short + island + argument condition. We only argue that in Lu et al.’s (2020) experiment there should be one condition in which the subject wh-phrase *shéi* ‘who’ rather than the object wh-phrase *shénme* ‘what’ is manipulated to occur inside an island so that the island effect on the subject wh-phrase can be tested.

4 Pragmatic considerations are not relevant to the English counterpart of (7) because wh-phrases in English undergo overt movement, and their overt movement out of a relative clause violates the syntactic island constraint. Of course, it can be said that pragmatic factors help to reduce the acceptability of sentences like (i), but they, not being a main factor, can be ignored. (i) *What did Wallace meet a woman that hates? One piece of evidence in support of our assumption that the ungrammaticality of (i) results from a syntactic constraint rather than a pragmatic constraint is that we cannot provide an appropriate context to make (i) acceptable, which is different from (7). See also Sichel (2018).

5 Since all the participants in our experiments are students of English majors, the use of English translation can help the participants better understand how *wèishénme* should be interpreted.

6 We also analyzed the data with both participants and items as random effects. The results were similar to the ones where the participants were the only random effect. Moreover, following the suggestion of one reviewer, after the experiment we asked another group of participants to rate the difficulty of sentences, and added this factor to the regression model. The results were not affected. For the sake of simplicity, we reported the results where only the participants were considered as random effect. Furthermore, after we transformed the rating scores into *z*-scores, and analyzed them with the model introduced above, the results were not affected. Since Lu et al. (2020) use raw ratings to discuss the source of *wh*-in-situ argument-adjunct asymmetry, following them we also utilize raw ratings. As to the identification of outliers, we followed the common practice in considering the scores that were 2 standard deviations away from the mean as outliers. We also analyzed the data without clearing the outliers to see whether our results were affected. Without finding any significant difference, in the study we reported the results where the data were not trimmed.

7 In this experiment, we recruited 64 participants rather than 96 participants because this can make the number of participants close to the one in Lu et al.’s (2020) experiment. In so doing, we are able to demonstrate that the number of participants is not a factor that leads to the lack of argument/adjunct asymmetry in Lu et al.’s (2020) experiment.

8 Our post-experiment interview confirmed our prediction. Both participants acknowledged that they interpreted *wèishénme* as ‘for what.’

9 There comes a question: what is the nature of the island constraints. Are the islands syntactic constraints (Ross, 1967; Bošković, 2016) or processing difficulties caused by the limitation of cognitive resource (Kluender and Kutas, 1993; Hofmeister and Sag, 2010; Hofmeister et al., 2012)? Although our study is compatible with the syntactic approach, here we do not claim that the processing approach is infeasible. We leave this important topic for future research. For the debate about the nature of islands with the acceptability judgment experiments, see Hofmeister et al. (2012), Sprouse et al. (2012), Goodall (2015), and Abeilléa et al. (2020), among others.

10 The frequency of sentence structures does not affect our result. For example, some participants’ rating scores for non-island conditions are very high, but other participants’ rating scores of these conditions are not high. After the experiment, we had an interview with those whose rating scores of non-island conditions were not high. They reported that they rated them low because they seldom used the multiply embedded clauses or saw them in daily life. In other words, their frequency was low (see also Xiang et al., 2015). We divided the data into two parts based on the rating scores for the short + non-island conditions. In one group the rating scores for the short + non-island condition were 6 or 7, and in the other group, the scores for this condition were below 6. Analyzing the data with the regression model constructed in the study, we found that in both groups a significant interaction effect of Structure × Wh-Category × Length could be observed, and the argument *wh*-in-situ versus adjunct *wh*-in-situ asymmetry in terms of island sensitivity could also be noted. This argues strongly for the idea that the frequency of certain conditions will not affect our experiment results. The reason behind this phenomenon is that the frequency effects can be deducted with the logic of the factorial experiment design. See also Section “The logic of factorial design for isolating the island effects and the previous formal judgment study into *wh*-in-situ.”

11 To ascertain what kind of examples are adduced to support the assumption that argument *wh’s*-in-situ are not subject to island constraints, we examined all the examples used in Aoun and Li (1993b), Tsai (1999), Huang et al. (2009), and Huang (2010), four classic and influential books that perform detailed studies of *wh’s*-in-situ. In these books, there are some examples repeated for sake of exposition. After the repeated examples were excluded, we collected 26 acceptable sentences where an argument *wh*-in-situ is located in a relative clause island/complex NP island, and among these sentences, 21 sentences involve subject *wh’s*-in-situ.

## References

[B1] Abeilléa A. Hemfortha B. Winckela E. Gibson E. (2020). Extraction from subjects: Differences in acceptability depend on the discourse function of the construction. *Cognition* 204:104293. 10.1016/j.cognition.2020.104293 32731004

[B2] Almeida D. (2014). Subliminal wh-islands in Brazilian Portuguese and the consequences for syntactic theory. *Rev. ABRALIN* 13 55–91.

[B3] Aoun J. Li Y. H. A. (1993a). Wh-Elements in Situ: Syntax or LF? *Linguistic Inq.* 24 199–238.

[B4] Aoun J. Li Y. H. A. (1993b). *Syntax of Scope.* Cambridge, MA: MIT Press.

[B5] Ariel M. (2019). Different prominences for different inferences. *J. Pragm.* 154 103–116. 10.1016/j.pragma.2019.07.021

[B6] Atkinson E. Apple A. Rawlins K. Omaki A. (2016). Similarity of wh-phrases and acceptability variation in wh-islands. *Front. Psychol.* 6:2048. 10.3389/fpsyg.2015.02048 26793156 PMC4709560

[B7] Bošković Ž (2016). On the timing of labeling: Deducing comp-trace effects, the subject condition the adjunct condition, and tucking in from labeling. *Linguistic Rev.* 33 17–66.

[B8] Cheng L. S. (2009). Wh-in-Situ, from the 1980s to Now. *Lang. Linguist. Compass* 3 767–791. 10.1111/j.1749-818X.2009.00133.x

[B9] Goodall G. (2015). The D-linking effect on extraction from islands and nonislands. *Front. Psychol.* 5:1493. 10.3389/fpsyg.2014.01493 25601844 PMC4283514

[B10] Hofmeister P. Casasanto L. Sag I. (2012). How do individual cognitive differences relate to acceptability judgments? A reply to Sprouse, Wagers, and Phillips. *Language* 88 390–400.

[B11] Hofmeister P. Sag I. (2010). Cognitive constraints and island effects. *Language* 86 366–415. 10.1353/lan.0.0223 22661792 PMC3364522

[B12] Huang C. T. (1982a). *Logical Relations in Chinese and the Theory of Grammar.* Cambridge, MA: MIT dissertation.

[B13] Huang C. T. (1982b). Move Wh in a language without Wh-movement. *Linguistic Review* 1 369–416. 10.1515/tlir.1982.1.4.369

[B14] Huang C. T. (2010). *Between Syntax and Semantics.* New York, NY: Routledge.

[B15] Huang C. T. Li Y. H. Li Y. F. (2009). *The syntax of Chinese.* Cambridge: Cambridge University Press.

[B16] Keshev M. Meltzer-Asscher A. (2019). A processing-based account of subliminal wh-island effects. *Nat. Lang. Linguistic Theory* 37 621–657. 10.1007/s11049-018-9416-1

[B17] Kluender R. Kutas M. (1993). Subjacency as a processing phenomenon. *Lang. Cogn. Process* 8 573–633. 10.1080/01690969308407588

[B18] Kush D. Dahl A. (2022). L2 transfer of L1 island insensitivity: The case of Norwegian. *Sec. Lang. Res.* 38 315–346.

[B19] Kush D. Lohndal T. Sprouse J. (2018). Investigating variation in island effects: a case study of Norweigian wh-extraction. *Nat. Lang. Linguistic Theory* 36 743–779. 10.1007/s11049-017-9390-z 30214096 PMC6130316

[B20] Kush D. Lohndal T. Sprouse J. (2019). On the island sensitivity of topicalization in Norwegian: An experimental investigation. *Language* 95 393–420. 10.1353/lan.2019.0051 34409987

[B21] Kuznetsova A. Brockhoff P. Christensen R. (2017). lmerTest Package: Tests in linear mixed effects models. *J. Stat. Softw.* 82 1–26.

[B22] Lasnik H. Saito M. (1992). *Move Alpha: Conditions on Its Application and Output.* Cambridge, MA: MIT Press.

[B23] Lu J. Y. Thompson C. Yoshida M. (2020). Chinese Wh-in-situ and islands: A formal judgment study. *Linguistic Inq.* 51 611–623. 10.1162/ling_a_00343

[B24] Michel D. (2014). *Individual Cognitive Measures and Working Memory Accounts of Syntactic Island Phenomena.* San Diego, CA: University of California dissertation.

[B25] Pañeda C. Lago S. Vares E. Veríssimo J. Felser C. (2020). Island effects in Spanish comprehension. *Glossa J. Gen. Linguistics* 5 1–30. 10.5334/gjgl.1058

[B26] R Core Team (2021). *R: A Language and Environment for Statistical Computing.* Vienna: R Foundation for Statistical Computing.

[B27] Ross J. (1967). *Constraints on Variables in Syntax.* Cambridge, MA: MIT dissertation.

[B28] Sichel I. (2018). Anatomy of a counterexample: Extraction from relative clauses. *Linguistic Inq.* 49 335–378. 10.1162/LING_a_00275

[B29] Sprouse J. (2007). *A Program for Experimental Syntax: Finding the Relationship between Acceptability and Grammaticality Knowledge.* College Park: University of Maryland dissertation.

[B30] Sprouse J. Caponigro I. Greco C. Cecchetto C. (2016). Experimental syntax and the variation of island effects in English and Italian. *Nat. Lang. Linguistic Theory* 34 307–344. 10.1007/s11049-015-9286-8

[B31] Sprouse J. Fukuda S. Ono H. Kluender R. (2011). Reverse island effects and the backward search for a licensor in multiple wh-questions. *Syntax* 14 179–203. 10.1111/j.1467-9612.2011.00153.x

[B32] Sprouse J. Hornstein R. (2013). *Experimental Syntax and Island Effects.* Cambridge, MA: Cambridge University Press.

[B33] Sprouse J. Wagers M. Phillips C. (2012). A test of the relation between working memory capacity and syntactic island effect. *Language* 88 82–123. 10.1353/lan.2012.0004 34409987

[B34] Stepanov A. Mušiè M. Stateva P. (2018). Two (non-) islands in Slovenian: A study in experimental syntax. *Linguistics* 56 435–476.

[B35] Tsai W. T. (1994). On nominal islands and LF extraction in Chinese. *Nat. Lang. Linguistic Theory* 12 121–175. 10.1007/BF00992747

[B36] Tsai W. T. (1999). *On Economizing the Theory of A-Bar Dependencies.* London: Routledge.

[B37] Tsai W. T. (2008). Left periphery and how-why alternations. *J. East Asian Linguistics* 17 83–115. 10.1007/s10831-008-9021-0

[B38] Warrena T. Gibson E. (2002). The influence of referential processing on sentence complexity. *Cognition* 85 79–112. 12086714 10.1016/s0010-0277(02)00087-2

[B39] Xiang M. Wang S. P. Cui Y. L. (2015). Constructing covert dependencies—The case of Mandarin wh-in-situ dependency. *J. Mem. Lang.* 84 139–166. 10.1016/j.jml.2015.05.006

