# Supplementary material for: Mandarin Chinese wh-in-situ argument–adjunct asymmetry in island sensitivity: Evidence from a formal judgment study
Source: Front Psychol. 2022 Sep 9;13:954175. doi: 10.3389/fpsyg.2022.954175 (PMC9505917; doi:10.3389/fpsyg.2022.954175)
Supplement: Supplementary file 1 [file Data_Sheet_1.pdf]

Table 1. the mean score and z-score of each condition in Experiment 1

Condition	Mean(SD)	Z-score(SD)
Short+nonisland+argument	4.94(2.00)	-0.09(0.93)
Long+nonisland+argument	4.75(1.98)	-0.18(0.92)
Short+island+argument	6.56(0.95)	0.66(0.44)
Long+island+argument	6.14(1.50)	0.46(0.70)
Short+nonisland+adjunct	5.95(1.48)	0.38(0.69)
Long+nonisland+adjunct	4.07(2.09)	-0.50(0.97)
Short+island+adjunct	6.53(1.03)	0.65(0.48)
Long+island+adjunct	2.20(1.61)	-1.37(0.75)

Table 2: Statistic summary of the three-way interactions in Experiment 1

	Beta	SE	t	p
Intercept	2.20	0.10	22.37	<.001
Structure	1.87	0.14	13.69	<.001
Length	4.34	0.11	40.01	<.001
Wh-Category	3.94	0.12	31.88	<.001
Structure × Length	-2.46	0.15	-16.02	<.001
Structure × Wh-Category	-3.26	0.17	-19.23	<.001
Length × Wh-Category	-3.91	0.15	-25.52	<.001
Structure × Wh-Category × Length	2.23	0.22	10.29	<.001

Model: score ~ Length * Structure * Category + (1 + Structure * Category | participant)

Table 3: Statistic summary of the interactions between Structure and Length when the wh's-in-situ serve as adjuncts in Experiment 1

	Beta	SE	t	p
Intercept	2.20	0.14	15.37	<.001
Structure	1.87	0.18	10.22	<.001
Length	4.34	0.16	26.66	<.001
Structure × Length	-2.46	0.19	-12.81	<.001

Model: score ~ Length * Structure + (1 + Length * Structure | participant)

Table 4: Statistic summary of the interactions between Structure and Length when the wh's-in-situ serve as arguments in Experiment 1

	Beta	SE	t	p
Intercept	6.14	0.10	63.24	<.001
Structure	-1.39	0.16	-8.85	<.001
Length	0.42	0.11	4.03	<.001
Structure × Length	-0.23	0.15	-1.52	0.13

Model: score ~ Length * Structure + (1 + Structure | participant)

Table 5: the mean score and z-score of each condition in Experiment 2

Condition	Mean(SD)	Z-score(SD)
Short+nonisland+argument	6.04(1.65)	0.22(0.79)
Long+nonisland+argument	5.89(1.69)	0.15(0.81)
Short+island+argument	6.55(1.17)	0.47(0.56)
Long+island+argument	6.15(1.25)	0.27(0.60)
Short+nonisland+adjunct	6.42(1.27)	0.40(0.61)
Long+nonisland+adjunct	4.84(2.13)	-0.36(1.03)
Short+island+adjunct	6.67(0.98)	0.53(0.47)
Long+island+adjunct	2.10(1.67)	-1.68(0.80)

Table 6: Statistic summary of the three-way interactions in Experiment 2

	Beta	SE	t	p
Intercept	2.10	0.13	16.43	<.001
Structure	2.74	0.17	16.03	<.001
Length	4.58	0.13	34.48	<.001
Wh-Category	4.05	0.14	29.35	<.001
Structure × Length	-2.99	0.17	-17.91	<.001
Structure × Wh-Category	-3.01	0.18	-16.48	<.001
Length × Wh-Category	-4.17	0.17	-24.91	<.001
Structure × Wh-Category × Length	2.74	0.24	11.59	<.001

Model: score ~ Length * Structure * Category + (1 + Length + Structure * Category | participant)

Table 7: Statistic summary of the interactions between Structure and Length when the wh's-in-situ serve as adjuncts in Experiment 2

	Beta	SE	t	p
Intercept	2.10	0.15	13.91	<.001
Structure	2.74	0.17	16.04	<.001
Length	4.58	0.17	26.60	<.001
Structure × Length	-2.99	0.17	-17.99	<.001

Model: score ~ Length * Structure + (1 + Length + Structure | participant)

Table 8． Statistic summary of the interaction effects between Structure and Length when the wh's-in-situ serve as arguments in Experiment 2

	Beta	SE	t	p
Intercept	6.15	0.11	57.56	<.001
Structure	-0.26	0.17	-1.55	0.13
Length	0.41	0.12	3.53	<.001
Structure × Length	-0.25	0.16	-1.62	0.11

Model: score ~ Length * Structure + (1 +Length + Structure | participant)
